# Comment on ‘Neuromuscular Impairment at Different Stages of Human Sarcopenia’ by Sarto et al.

**DOI:** 10.1002/jcsm.13624

**Published:** 2024-10-20

**Authors:** Ross A. Jones, Abdullah Ramadan, Shahd Qutifan, Thomas H. Gillingwater

**Affiliations:** ^1^ Edinburgh Medical School: Biomedical Sciences University of Edinburgh Edinburgh UK; ^2^ Euan MacDonald Centre for Motor Neuron Disease Research University of Edinburgh Edinburgh UK

We read with interest the paper entitled ‘Neuromuscular impairment at different stages of human sarcopenia’ by Sarto et al. [1], which has used a variety of assessment approaches to innovatively investigate neuromuscular impairment in older human subjects. This study undoubtedly represents an important contribution to our understanding of the impact of age and sarcopenia on the human neuromuscular system, for which we wish to congratulate and thank the authors.

Although the authors have presented a lot of important new data, they state in their conclusions that ‘an altered innervation profile and NMJ instability are prominent features of the ageing of the neuromuscular system’. This is based on the claim that an increase in neural cell adhesion molecule‐positive fibres is evidence of ‘increased muscle denervation’. We would like to raise caution against such a conclusion when based on the type of indirect data presented by Sarto et al. [1].

The term ‘muscle denervation’ refers to a specific process whereby the nerve supply (innervation) of a muscle fibre, derived from a lower motor neuron input, is removed or lost. For this term to be accurate, therefore, it requires evidence showing that lower motor neuron inputs at the NMJ are removed or lost. We would politely suggest that an increase in neural cell adhesion molecule‐positive fibres is not such evidence. Rather, anatomical and morphological studies of the NMJ are required to be able to draw such conclusions.

Sarto et al. [1] point out in their paper that morphological evidence for NMJ disruption, and hence muscle denervation, is present in rodent models of ageing [2] and use this to support their claims in humans. However, whilst technically challenging, comparable morphological studies of NMJs in young and old humans have been performed, showing that, in stark contrast to rodent models, NMJs remain structurally intact over the normal human lifespan [3]. Such species‐specific differences are perhaps not surprising given the inherent differences that exist in NMJ structure and stability between rodents and humans [4, 5]. As such, the strongest *direct* evidence available to date suggests that motor neuron inputs at the NMJ are not removed or lost with increasing age in humans. This in no way precludes the possibility that age‐ and/or sarcopenia‐related changes, such as those reported by Sarto et al., [1] are occurring in muscle that mimic denervation‐induced changes. Rather, it questions the premise that such changes are occurring due to a loss of innervation resulting from structural breakdown of the NMJ.

The use of indirect measurements to draw conclusions about NMJ status and muscle denervation is not an issue solely restricted to Sarto et al.'s paper [1], and we by no means wish to single out this important study in this regard (indeed, we strongly welcome the newly presented evidence of age‐related functional decline in the human neuromuscular system). Understandably, many studies are not in the position to obtain fresh muscle biopsies containing NMJs suitable for morphological assessment, for both technical and/or logistical reasons. In our own experience, obtaining such samples is a frustrating, time‐consuming and complex task. However, we would contend that experiments facilitating direct morphological assessment of the NMJ are required for studies to be able to draw robust conclusions concerning muscle denervation and NMJ status.

We hope that these comments will be useful for colleagues considering the design and reporting of future studies in this field.

## Conflicts of Interest

The authors declare no conflicts of interest.
